# An Unexpected Complication During Transaortic Valve Replacement

**DOI:** 10.7759/cureus.11631

**Published:** 2020-11-22

**Authors:** Diego Celli, Jason Galo, Cesar E Mendoza

**Affiliations:** 1 Internal Medicine, University of Miami Miller School of Medicine, Miami, USA; 2 Cardiovascular Disease, Jackson Memorial Hospital, Miami, USA

**Keywords:** aortic valve disease, vascular air embolism, new generation tavr

## Abstract

Venous air embolism is a rare procedural complication with devastating consequences depending on the amount of air introduced into the body. Herein we present a case of transaortic valve replacement complicated with air embolism leading to cardiopulmonary collapse successfully treated with mechanical circulatory support.

## Introduction

Transaortic valve replacement was once the treatment of choice only for severe aortic stenosis patients deemed to be at high surgical risk or inoperable; its indications have since been expanded to include moderate and low-risk patients. While evidence has shown non-inferiority versus surgical aortic valve replacement, it has intrinsic complications including device migration, paravalvular leak, myocardial infarction, acute kidney injury, stroke, vascular access adverse events, and even death.

Venous air embolism occurs when air enters the venous system due to a pressure gradient between the site of air entry and the right atrial pressure. Significant air embolism may occur during the insertion or removal of large-bore venous catheters and the administration of pressurized intravenous infusions. Although minor cases are associated with minimal or no symptoms, significant volumes can result in acute right-sided heart failure with hemodynamic instability and cardiac arrest.

## Case presentation

A 75-year-old female with a history of chronic kidney disease stage III, heart failure with preserved ejection fraction New York Heart Association (NYHA) Functional Classification Class III Stage C, and symptomatic severe aortic stenosis Stage D1 presented to our institution to undergo a scheduled Transaortic Valve Replacement (TAVR). Pre-procedural transthoracic echocardiogram revealed a preserved left ventricular systolic function (ejection fraction 60-65%), aortic valve area of 0.83 cm2, increased maximum aortic velocity of 3.8 m/s, abnormal transvalvular gradients with a mean pressure gradient of 50.5 mmHg, and stroke volume index of 37.2 ml/m2. There was evidence of mild mitral regurgitation, but no other significant valvular disease. The right ventricular systolic pressure was 50-60 mm Hg and the right ventricle size and function were normal. Cardiac catheterization confirmed the echocardiographic hemodynamic assessment of the aortic valve. In addition, coronary angiography showed mild non-obstructive coronary artery disease. A computed tomographic (CT) scan demonstrated a bicuspid aortic valve and suitable anatomy for a transfemoral approach.

During the cardiac procedure, after the temporary pacemaker lead and all the large bore sheets were put in place, the anesthesiology team requested to connect a high flow extension line to the side port of the venous femoral sheath. Unfortunately, an inadvertent air flush of an unknown volume was administered through the above-mentioned line resulting in a sudden hemodynamic compromise with rapidly progressive severe hypotension and tachycardia ending up in pulseless electrical activity (PEA). Advanced resuscitation maneuvers were initiated obtaining the return of spontaneous circulation after five minutes.

**Figure 1:**
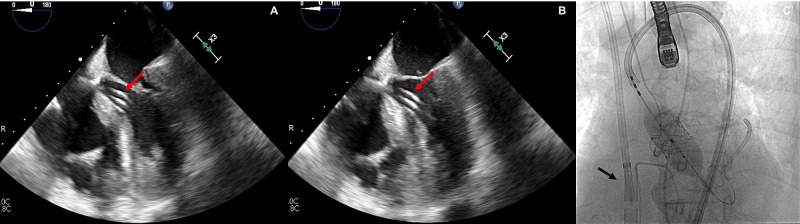
Intraprocedural transesophageal echocardiogram showing right ventricular global dysfunction with an Impella® left ventricular assistance device (red arrow) in the left ventricle. A) Diastole B) Systole. C) Fluoroscopy image depicting balloon valve inflation in-place with the extracorporeal membrane oxygenation system (ECMO) cannula in venous system (black arrow).

Given her hemodynamic instability, an Impella® left ventricular assistance device had to be inserted and she was placed on high inotropic and vasopressor support to maintain arterial pressure and organ perfusion. Despite all these measures, the patient’s condition deteriorated further and she had another brief episode of PEA. At this point, a transesophageal echocardiographic assessment demonstrated acute right ventricular failure (Figure 1 A and B). Therefore, we decided to escalate her hemodynamic support with a percutaneously implanted venous-arterial extracorporeal membrane oxygenation system (ECMO) (Figure 1 C).

The patient experienced significant improvement overnight with a meaningful recovery of her right ventricular function; therefore, the heart team decided to perform TAVR the following day. This was done uneventfully after removing the Impella® device and while retaining ECMO support. An Edwards SAPIEN 23 mm valve was implanted, and optimal hemodynamic parameters were noted subsequently. This allowed for the discontinuation of mechanical circulatory support. She was managed in the intensive care unit to monitor complications for ten days; given her significant deconditioning she was transferred to the acute inpatient rehabilitation facility where she recovered successfully. She has scheduled cardiology outpatient follow-ups.

## Discussion

It is estimated that the amount of venous air embolism required to cause circulatory collapse and death in humans is around 100-300ml. In less than half a second, a 15French sheath can allow 300 ml of air to enter the vasculature; circulatory collapse occurs when this bolus of air enters the right ventricular outflow tract (RVOT). Some experts believe that the churning action of the heart creates a foamy mass of blood mixed with air and fibrin, which causes a mechanical obstruction in the RVOT. It has also been suggested that pulmonary hypertension can contribute to circulatory collapse when air and fibrin enter the pulmonary arterioles [1].

The early clinical symptoms and signs in non-sedated patients are non-specific and include dyspnea, chest pain, vomiting, and a sense of impending doom. In patients under anesthesia, such as our patient, reduced end-tidal CO2, tachycardia, and hypoxemia are early clinical manifestations. When the circulatory collapse happens, it can lead to hypotension, PEA, and cardiac arrest. ECG may demonstrate ST-segment changes and evidence of right heart strain. Echocardiography is useful to demonstrate right ventricular dysfunction in these cases. On rare occasions, chest CT scans can detect small amounts of air in the right ventricle or pulmonary vasculature [1].

Prevention of venous air embolism is a matter of paramount importance. Strategies to increase the central venous pressure, which decreases the gradient between the right atrium and atmospheric pressure are among the first steps to be taken. These include treating hypovolemia, placing patients in the Trendelenburg position, and avoiding inserting the catheters during patient inspiration. In addition, all catheter lumens should be flushed before placement, and the handling of pressurized infusions must be done with extreme caution to prevent air entry into the body [2]

Management of venous air embolism includes prompt initiation of 100% high-flow oxygen to facilitate the diffusion of nitrogen, which decreases the size of the embolism. Specific positions like the Durant and Trendelenburg positions may mitigate RVOT obstruction by facilitating the trapping of air in the apex of the right ventricle and into the right atrium. If circulatory collapse occurs, it is important to initially address the ABCs (airway, breathing, and circulation), and if necessary, prompt initiation of cardiopulmonary resuscitation (CPR). If the patient is unconscious, closed-chest compressions may help to force the air into small pulmonary vessels, relieving the obstruction. A right atrial catheter can also be employed to aspirate the trapped air. Other advanced therapies such as extracorporeal membrane oxygenation (ECMO) should be considered promptly [2]

In the present case, we suspect that the amount of air introduced in the system was large enough to cause right ventricular failure and PEA due to RVOT or pulmonary vasculature obstruction. This was successfully managed with mechanical circulatory support ultimately allowing the patient to undergo her TAVR procedure.

## Conclusions

Our experience delineates a case of an adverse event related to inadvertent venous administration of an unknown amount of air during a transaortic valve replacement leading to pulseless electrical activity. Vessel catheterization is routinely done and needs to be handled mindfully to prevent air embolism and its potentially devastating consequences. Prompt recognition is of utmost importance to initiate the appropriate treatment.
